# Flipped classroom as a reform-oriented approach to teaching mathematics

**DOI:** 10.1007/s11858-020-01191-5

**Published:** 2020-10-07

**Authors:** Mustafa Cevikbas, Gabriele Kaiser

**Affiliations:** 1 Universität Hamburg, Faculty of Education, Von-Melle-Park 8, 20146 Hamburg, Germany; 2 Australian Catholic University, Institute for Learning Sciences and Teacher Education, Brisbane, Australia

**Keywords:** Flipped classroom, Mathematics teaching, Reform in teaching mathematics, Teaching practices

## Abstract

Innovative methods can change the paradigm of teaching mathematics and inspire teachers to espouse new ideas and gain new experiences. The flipped classroom (FC) is currently an innovative pedagogical approach that has high potential to transform the teaching of mathematics. In the case study described in this paper, we investigated one mathematics teacher’s transformation of teaching in two mathematics classrooms through implementing interventions based on FC methods; furthermore, we identified several key points of FC design as well as challenges and opportunities afforded by teaching mathematics in FCs. The results of the study showed that the tasks posed by the teacher, the implemented discourse, teacher feedback and scaffolding, and the teaching–learning environment were changed in FCs, although the approaches used by the teacher to analyze the tasks and students’ learning were similar to those used in non-FCs, which points out the strengths of traditional teaching approaches. The study indicates that although teaching mathematics in FCs created some difficulties for teaching, well-designed FCs offered a great opportunity to promote students’ mathematical thinking and understanding. Overall, the results highlight that through FC, teachers can develop students’ mathematical potential with FCs.

## Introduction

Digital technologies have the potential to change the content of school mathematics as well as to foster the development of mathematical knowledge and understanding (Heid 2005; Olive et al. 2009). New technologies present different ways to interpret communication, collaboration, and social interaction (Beatty and Geiger 2009), and encourage a strong connection between mathematical knowledge and practice (Olive et al. 2009). Educators have attempted to create and familiarize themselves with technological artefacts to enhance mathematics education (Lagrange and Kynigos 2014). While these attempts have the power to change classrooms, research into how this change can be accomplished and its actual implementation frequently lag behind the speed of the digital evolution (Goos et al. 2020). The rapid dissemination of technology use in society has not reached education fully, at least before Covid-19 technology had still a rather marginal status in mathematics teaching and learning (Lavicza 2010). Hence, there is an increasing desire among mathematics teachers to use technology-enriched teaching approaches, consider the use of technology as part of their teaching activities, access resources, and share knowledge and experience with their colleagues (Hooks 2015; Joubert et al. 2020).

Employing technology-supported reform-oriented approaches in mathematics teaching has become a necessity for the twenty-first century, even before the changes forced by the pandemic Covid-19 came into the public discussion. We can define the term of reform as “change or changes made to a system or organization in order to improve it” (Longman 2020). The flipped classroom (FC) is one of the reform-oriented approaches that can accelerate digital transformation in teaching mathematics, contribute to technology integration into mathematics education, and engage students in mathematics. FC is described as a teaching reform (He 2020) that changes teaching ideas, objectives of teaching, teaching time, and teaching mode with the help of technology (Jian 2020) in such a way that within FC instructors teach students in-class, and learners do homework by themselves at home (He 2020). FC is defined on first access as “school work at home and homework at school” (Bergmann and Sams 2012). Lecture videos, notes, slides, and articles are shared with students outside of the classroom, and teachers have an opportunity to communicate and interact with their students by means of online learning management systems (LMSs), and then support deep learning through face-to-face classroom activities. Flipping the teaching provides more time for active learning and problem-solving activities in the classroom (Lo and Hew 2017). McGivney-Burelle and Xue (2013) describe the main principles of FC as follows: (1) out-of-class time should be well-structured to prepare learners for class hours (2) teachers assess learners’ pre-class activities (3) class time consists of collaborative and cooperative problem-solving activities and discussions, and (4) well-structured and highly organized implementation of a learning environment is based on scaffolding and feedback by the teacher. In essence, the FC approach has the potential to improve mathematics teaching and learning and use technology to accomplish these goals. Teachers should use technology strategically in such a way that all the students can access mathematics (National Council of Teachers of Mathematics 2011). FCs can provide this strategic use of technology and have the potential to improve mathematics teaching.

However, it appears that currently only a limited number of studies have examined the teaching experience of (secondary) mathematics teachers and their roles within the FC framework (Fredriksen 2020). The vast majority of studies in the FC context focus on students and their learning rather than teachers and teaching in (secondary) mathematics classrooms. In this study, we analyze the change of mathematics classrooms by FC from the perspective of the teacher. In addition, we identify the pros and cons of teaching mathematics in FCs. Overall, the study addresses the following research questions from the teachers’ perspective:

1. How does the flipped classroom transform mathematical teaching approaches?
2. What are the challenges and opportunities provided by teaching mathematics in the flipped classrooms?

## Background

### Concept of flipped classroom

Different names for FC pedagogy have been proposed so far, e.g., inverted classroom (Lage et al. 2000), classroom flip (Baker 2000), flipped classroom (Bergmann and Sams 2012). Furthermore, FC has been defined in different ways, e.g., by Lage et al. (2000) as “Inverting the classroom means that events that have traditionally taken place inside the classroom now take place outside the classroom and vice versa” (p. 32), although other definitions point out that FC is going beyond this approach. Bishop and Verleger (2013) offered a definition for FC that is composed of two parts, namely, interactive group work in the classroom, and computer-based instruction out of the classroom, including lecture videos. One of the more current and comprehensive definitions of FC has been developed by the Flipped Learning Network (FLN). According to this definition:

FC is a pedagogical approach in which direct instruction moves from the group learning space to the individual learning space, and the resulting group space is transformed into a dynamic, interactive learning environment where the educator guides students as they apply concepts and engage creatively in the subject matter. (FLN 2014).

A group of educators from the FLN suggested that teachers should incorporate four pillars (F-L-I-P) into their teaching practice, creating the widely used acronym FLIP:

1. *F: Flexible environment:* Teachers should rearrange the teaching–learning environment to adapt to each lesson or unit to encourage either independent or group work. These newly created environments empower students to select the desired learning time and place.
2. *L: Learning culture:* In FC, class time should be devoted to inquiring, learning subjects and content-specific concepts more deeply, and generating learning opportunities. Teachers in FC make use of scaffoldings to enable their students to find out specific topics by thorough implementation of student-centered approaches in the zone of their proximal development (Vygotsky 1978).
3. *I: Intentional content:* Teachers should constantly consider how they could benefit from the FC approach to support learners in enhancing deeper understanding. Teachers need to decide on the content to be taught and materials the students should explore.
4. *P: Professional educator:* Although the visibility of the professional educator’s role is less obvious in FCs, teachers in this environment are much more important and frequently more challenged than in traditional classrooms. They should consistently observe students, provide support, give comprehensive feedback, and assess students’ work.

Although FC has been defined in different ways, there is a consensus that FC is a student-centered pedagogy, giving teachers more time for implementing active learning activities, enabling social interaction and collaboration, creating technology-rich environments in accordance with differentiated learning, and presenting opportunities for students to move through the zone of proximal development (for details see Cevikbas and Argün 2017). In FC, the learning and teaching process is not confined to the classroom; students can progress at their own pace in an interactive way both in and out of the classroom (Davies et al. 2013), and teachers can provide effective guidance for students rather than deliver information directly. In this study, the FC definition offered by the FLN (2014) was used, taking into account that lecturing videos are a crucial part of the FCs.

### Affordances of a FC in teaching mathematics

The current generation of students has quite different characteristics, expectations, and dispositions compared to students a few decades ago (Cevikbas and Argün 2017). Nowadays, students prefer to access information quickly, and, especially by using various digital technology channels, they desire to construct their own knowledge by enjoying themselves (Cevikbas 2018; Engelbrecht et al. 2020). Teaching methods and learning environments have to be adapted to respond to these changes. FCs provide students with a tailored learning environment by inverting traditional teaching approaches with the help of technology. In this way, FCs help to create high-quality mathematics teaching activities (Chen and Wen 2019) and can develop students’ learning opportunities in mathematics. FCs encourage students to enhance their critical thinking abilities, assist in clarifying the goals of learning collaboratively, and think about mathematics problems before participating in classroom activities (Mazur et al. 2015; Voigt et al. 2020). Teachers in FCs gain additional time to apply inquiry-based activities, problem-solving activities, hands-on activities and comprehensive analysis in their classrooms (Schmidt and Ralph 2016). They can spend class hours creatively and strategically and can interpret students’ mathematical thinking (Fulton 2012). The FC approach transforms mathematics classrooms into laboratories of inquiry, analytical thinking, and connectedness with other fields of STEM (Bergmann and Sams 2012).

While FCs benefit students by encouraging them to engage in mathematics from behavioral, emotional, and cognitive perspectives (Cevikbas and Argün 2017), they also benefit teachers by helping them to improve their professional competencies and transforming classroom dynamics. Radical changes in the teaching experiences generate persuasive ideas and authentic vision (Brown 2018). Due to the nature of FC, even if advanced use of technology is not required, teachers need at least basic competencies in using technology in mathematics teaching for implementing the FC approach. This requirement allows them to learn new technologies and new teaching strategies (Brown 2018). Teachers have an active role in FCs and can provide guidance and scaffolding to their students when they need professional support (Cevikbas and Argün 2017; FLN 2014). They can also follow students’ learning progress and offer timely and comprehensive feedback. FCs increase teacher–student interactions (Bergmann and Sams 2012; Brown 2018; Cevikbas and Argün 2017; Lo and Hew 2017) and alleviate disciplinary problems in the classroom (Cockrum 2014). They also change classroom management and make classrooms more transparent; that is FCs make it possible for parents to follow teachers and students activities (Bergmann and Sams 2012).

### Difficulties of teaching in flipped mathematics classrooms

Despite the numerous advantages of FCs in mathematics teaching, difficulties that teachers may encounter are reported in empirical studies. We can summarize the difficulties of flipped teaching along three basic categories: (1) *paradigm shift*, (2) *content*, and (3) *technical requirements*. The first difficulty refers to changing the pedagogical *paradigm* of teaching and learning mathematics (Cevikbas and Argün 2017; Lo and Hew 2017). Students’ and teachers’ beliefs and perceptions can create paradigmatic barriers to flipped teaching and learning. There is a potential risk in FCs that students may skip the out-of-class tasks and come to the classroom without watching the lecture videos due to lack of independent learning responsibility. Another difficulty is related to creating subject-specific *content*. Teachers need to have well-prepared lecture videos, notes, slides, and teaching materials to implement effective flipped teaching (Chen 2016; Lo and Hew 2017). In particular, it is difficult to find customized lecture videos or another type of content that effectively meets teachers’ and students’ needs and expectations. Although there are plenty of videos accessible on online platforms such as YouTube, Teacher Tube, Khan Academy, and so forth, existing videos do not cover or match all topics taught in school mathematics (Chen 2016). In this case, teachers have to create their own lecture videos, which is an extremely time-consuming task. Another difficulty for FC implementation is to have the *technical requirements* to teach and learn mathematics. The technical problems with accessing the Internet and mobile devices cannot be underestimated and may destroy the structure of FCs. FC practices do not work well without the Internet, and teachers should be able to use technology strategically in the process of teaching mathematics. Organizing and operating the resources, tasks, students, and knowledge simultaneously can create complicated problems for teachers who need to improve their technology use competencies (Trigueros et al. 2020). In addition, while flipping the classes, teachers may have to deal with some additional challenges, such as dissatisfaction, unwillingness, and bias against FC (Bagley 2020; Chen 2016).

### Theoretical framework

Many educators are still stuck with traditional teaching approaches, which are rooted within the paradigm of transfer of knowledge by lectures, notes, and presentations. These approaches simply concentrate on knowledge memorizing and do not encourage critical thinking, problem solving, collaboration, engagement, and social interaction (Marzouki et al. 2017). Contemporary educational approaches promote interactive teaching and learning environments based on technology. FC pedagogy is one of the most adapted innovative approaches for creating interactive teaching and learning environments, as it has the potential to enhance problem solving, collaboration, engagement, social interaction, and communication (Bergmann and Sams 2012; Cevikbas and Argün 2017). FC can improve teachers’ differentiated teaching experiences and allow them to guide and support their students in and out of the classroom. Overall, FC offers a relatively new teaching approach strongly connected to Vygotsky’s (1978) ideas based on social constructivist theory (Ahmed 2016; Jarvis et al 2014). From the social constructivist perspective, knowledge and meaning are socially constructed by interaction and higher-order cognitive teaching–learning activities in FC. Learning is described as the creation of an environment in which students are active in constructing their own knowledge (Schreiber and Valle 2013). According to the Vygotskian approach a good teacher will create an interactive and useful environment by fostering discovery and socialization (Kim 2001; Schreiber and Valle 2013). Vygotsky (1978) introduced the concept of zone of proximal development (ZPD) pointing out that learners can proceed to the next zone of their proximal development with the help of more knowledgeable individuals. Based on the theoretical framework of social constructivism, teachers are expected to perform the following tasks in FCs (Bergmann and Sams 2012; Cevikbas 2018): (1) design an interactive classroom environment (including virtual classrooms) and help students to prepare for class hours by use of videos and online resources; (2) support students to construct knowledge and meaning by providing scaffolding and sufficient feedback; (3) promote the agency of students to think, inquire, communicate, interact, and discuss; (4) design and implement activities that foster active learning for their students; and (5) use dynamic assessment approaches. When used as an educational tool, *technology* contributes to the development of social constructivist meaning by encouraging social interaction, communication, discussion, problem solving, engagement and collaboration. Therefore, social constructivism is one of the most adopted theories for technologically rich environments (Marzouki et al. 2017) and aligns with the benefits of FC pedagogy (Bishop and Verleger 2013; Jarvis et al 2014). Accordingly, we have embedded our FC research study within a social constructivist framework, which allows us to examine the changes of the mathematics teaching with FC interventions, determine the opportunities and challenges of flipped mathematics teaching, and construct an effective design of a FC for mathematics teaching.

## Methodological approach

### Research design

In this study, we employed qualitative research methods to investigate how mathematical teaching was changed by teacher’s FC interventions and explore opportunities and challenges inherent in flipped teaching. Qualitative research, especially case studies, are appropriate for the investigation of people’s knowledge, views, and experiences (Merriam and Tisdell 2016). Case studies focus on a bounded system by collecting data through multiple sources of information such as interviews, observations, audio-visual media, and documents (Creswell 2013). The most explicit examples of bounded systems are a single person, a group, an institution, or a subject (Merriam and Tisdell 2016). This study was designed as a qualitative case study, which is particularly appropriate for exploratory studies aiming for insight into flipped teaching experience and the design of FC, as well as the pros and cons of FCs in teaching mathematics. Our bounded system was a single mathematics teacher and we focused on her teaching experiences in two separate secondary classrooms.

### Participants and data collection

The voluntary participants of the study were a mathematics teacher and 68 high school students at a public school in Turkey. The school was located in a neighborhood where families with medium socio-economic background resided. The participating teacher was selected based on volunteering principles and is named in this study Ece (pseudonym). Ece had 6 years of professional experience as a mathematics teacher and at the time of study was continuing her doctoral studies in the field of mathematics education in Turkey. Although she was familiar with student-centered teaching methods from her undergraduate and graduate education, she mostly applied direct instruction methods in her classes and the students were used to learning mathematics through a teacher-centered approach. Ece taught mathematics in three separate secondary classes (9th grade, 10th grade, and 11th grade) for a total of 18 h per week. In this study, we focus on her 10th grade and 11th grade classes. Using basic digital technologies and having Internet-based devices and stable Internet connection at home were required for all students as well as the teacher, in order to conduct an effective FC implementation.

**Fig. 1:**
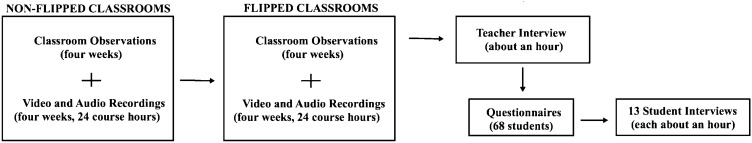
Data collection sequence of the study

The main data sources of this research study were classroom observations, video and audio recordings, semi-structured teacher and student interviews, and questionnaires. A significant part of case studies is the use of multiple data sources to prevent systematic faults and to resolve inconsistencies (Maxwell 2013). In this study, Ece’s teaching experience can be divided into two parts: Non-flipped teaching and flipped teaching. In the first part, Ece taught mathematics for 4 weeks in the non-FC settings and we (more precisely, the first author of the paper) observed her in two non-FCs (10th grade and 11th grade) and recorded her teaching experiences and classroom activities via audio and video recorders. Classroom activities and Ece’s teaching experiences in non-FCs were recorded with a video camera placed in the back corner of the classroom. In addition, Ece’s talks were recorded by a voice-recorder placed on the teacher’s desk, since Ece was generally close to the teacher desk while she was lecturing in non-FCs. In the second part, Ece flipped her mathematics classrooms for 4 weeks. She was observed in two FCs and her teaching experiences were recorded with a video camera. In FCs, Ece led group work, and walked around the classroom to guide students and follow their work most of the class time. In order to record the conversations between Ece and the students, voice-recorders were placed on each group's desks. After the 4-week FC experience, we gave 68 students a questionnaire consisting of 15 open-ended questions asking about their views on the teacher’s teaching approach in FCs and non-FCs. Then, we performed semi-structured interviews, each lasting about an hour, with the teacher and 13 students. For the selection of the students to be interviewed, the teacher’s opinions, students’ mathematics scores, and results in the questionnaires were used. We gathered the data from interviews about teaching approaches in FCs and non-FCs, opportunities and challenges of the FC approach, and the design of a FC. Figure 1 shows the data collection sequence of the study.

### Design of the teaching environment

First, the participating teacher was offered individual professional development concerning the FC approach. This professional development consisted of papers and teaching material to enlarge her knowledge about FC design and implementation. Then, with the support of the first author, the teacher developed the FC design and created the related content. Lecture videos and notes, worksheets, and classroom activities related to polynomials and logarithms were prepared using online sources and audiovisual or text-based materials. Three independent mathematics educators evaluated the appropriateness of the teaching materials that were prepared for the FC implementations. A single virtual classroom for the pilot study and two virtual classrooms for the main study were created using the Edmodo LMS (https://new.edmodo.com/). The pilot study was conducted with the same teacher and 10th-grade students for 2 weeks based on two lecture videos shared on the learning platform. We observed the teacher’s performance in the pilot study and made some adjustments regarding the videos and the planned classroom activities. Additionally, the teacher was able to gain some teaching experience with flipped teaching before the main study. In the pilot study, we realized that the teacher had difficulties in managing the group work due to the crowded classroom and the high number of students in the class. We also realized that lecture videos should not be shared too early (for example, a week before) or too late (for example, a day before) before having the associated meeting in the classroom. Based on the teacher’s and the students’ feedback, the appropriate length of the lecture videos was determined to be between 10 and 20 min and should include examples, problems, solutions, prompt questions, and, of course, lecturing. Explanations about the basic structures of FC design adopted in this research are summarized in Table 1.

**Table 1:** Overview of design elements of FC

Environment	Activities and Tasks	Time
Out of class	Lecture offering	10–20 min per each video
	Taking notes from the lecture (for students)	5–10 min
	Q&A on lecture video (teacher or students could ask and reply to questions whenever they wanted on LMS)	Flexible
	Searching for additional source(s)	Flexible
In class (over 40 min)	A brief summary of video lecture	5 min
	Q&A about lecture offered out of class	5–10 min
	Active learning activities under the guidance of the teacher	20–25 min
	Information about next lesson/lecture video	3–5 min

After the pilot study, observations were conducted lasting 4 weeks (24 course-hours with each course-hour lasting 40 min) in traditionally organized classes (10th and 11th grades) where the teacher taught mathematics. The lessons were recorded by video camera in order to evaluate the teacher’s usual teaching approach and the usual teaching atmosphere within the traditional teaching environment. Immediately afterwards, FC was implemented for 4 weeks (24 course-hours) in both of the classrooms. Six lecture videos for the 10th-grade students and four lecture videos for the 11th-grade students were uploaded to the learning platform at least 2 days before the associated lessons. FC activities were applied in accordance with the design described in Table 1. In-class activities were observed and recorded by video cameras and voice-recorders. Teacher and students were interviewed and questionnaires were applied in the fourth week of the FC implementations.

### Data analysis

In this case study, our bounded system consisted of a single mathematics teacher and we focused on her teaching experiences in both FCs and non-FCs. In the data analysis process, firstly we watched the 48 video recordings and listened to the 26 audio recordings (interviews and classroom recordings) one more time and transcribed them verbatim. Then, we read the transcribed data from video-audio recordings, the completed questionnaires consisting of open-ended questions about the teaching approach of the teacher, and observation notes several times. Then, we encoded the data through the content analysis method (Miles and Huberman 1994) and identified four categories for the first research question: (1) *environment,* (2) *interaction*, (3) *feedback* and *scaffolding* and, (4) *assessment*. We determined differences between teaching in flipped and non-flipped classrooms in terms of these themes that are important in teaching based on social constructivism (Marzouki et al. 2017; Palincsar 1998; Vygotsky 1978). In social constructivism, knowledge is constructed by means of social interaction, communication and environment, in other words learning should not be considered in isolation from the environment and socialization (Kim 2001). Learners can proceed in their ZPD with scaffolding and specific supportive types of feedback given by teachers (Cevikbas and Argün 2017). Thurlings et al. (2013) conducted a review study and identified timing, characteristics and effects of FC as characteristic features based on social constructivism as follows: (a) *timing* (immediately, when students are reminded of their actions/when still relevant), (b) *characteristics* (focused on/related to task, related to goal and student’s perceptions of their performance; includes information about progress, targets at a suitable level, gives opportunity to respond, dialogue/context of collaboration, encourages positive motivational beliefs and builds empathy), and (c) *expected effects* (supports learner to engage in action to close gap, engages students in thinking) of feedback in social constructivist theory. Table 2 indicates some examples of the coding related to the feedback provided. According to the social constructivist approach, scaffolding is a temporary support provided by an expert who helps a learner understand how to perform a similar task that s/he may encounter in the future (Wood et al. 1976) and assessment is referred to as dynamic assessment that provides a future measure of performance, signifying capacity. This capacity provides information about how the learner will perform individually in the future (Palincsar 1998). We employed content analysis (Miles and Huberman 1994) to ascertain challenges and opportunities inherent in the flipped mathematics teaching. Finally, two themes (1) *challenge*, and (2) *opportunity* for the second research question could be identified. Since we did not aim to compare the results related to mathematics teaching experience between the 10th grade and the 11th grade students, we analyzed the data collected from both groups together. After coding, code frequencies and quotations backing the results were identified. We used a code–recode strategy in which we recoded 30% of the research data after an interval of 6 weeks and achieved a consistency rate of 99% between these two encodings.

**Table 2:** Examples of coding of data concerning teaching in mathematics classrooms

Theme	Category	Code
Feedback	Timing	Immediate feedback, delayed feedback, feedback is given at the beginning of the lesson, feedback is given at the end of the lesson, feedback is given when students perform group work, feedback is given out of the lesson
	Type	Process-oriented feedback, i.e., feedback consists of hint, explanation, support, elaboration, inquiry, encourage Result-oriented feedback, i.e., corrective, confirmative, evaluative Based on source: teacher, peer
	Frequency	Number of feedback instances given in a lesson

Furthermore, an additional mathematics educator carried out double coding of 10% of the data. The rate of intercoder reliability was computed using Miles and Huberman’s (1994) formula and was determined to be 92%. We examined our codes and worked on them until we achieved total reliability. We shared our pre-results with the teacher, and she confirmed our results. The member check strategy allowed us to reduce some misinterpretations, errors, and prejudices (Maxwell 2013).

## Results of the study

### Transformation in teaching mathematics from the teacher’s perspective

For this study, the results of the transformation that FC interventions created in mathematics teaching are presented in four groups; *teaching and learning environments, interaction and communication, teacher feedback and scaffolding,* and *assessment strategies*.

#### Teaching and learning environments

Before the intervention, Ece had taught in non-FCs by standing in front of the blackboard or sitting at the teacher desk. She used the direct instruction technique and asked her students to write down what she had explained or written on the blackboard. Although there was a smartboard in the classroom, Ece preferred to use the blackboard in her lessons. The classroom observations of traditional teaching by Ece revealed that she did not draw on technology or concrete materials in order to enhance the understanding of the students. Ece lectured based on only one course book and did not tailor her teaching to the knowledge, understanding, and experiences of the students; in contrast, she treated them as passive listeners. Ece did not assign her students tasks based on mathematical activities, and she did not conduct problem-solving activities in her traditional teaching. The student desks were located in a way that did not allow for group work, which had not been carried out before in the classes involved. We could observe in the non-FCs that while Ece was lecturing, the students from the back rows were engaging in some disruptive behaviors (playing games on smartphones, eating sunflower seeds, talking to peers, listening to music, etc.). It became obvious that the structure of the classes (being crowded, desks arranged in rows) and the teaching approach that Ece had adopted did not provide opportunities for active learning. Ece explained the change in mathematics classes through FC as follows:

Ece: *In my ordinary classes, the time is already limited so I used the direct instruction method. I could not construct a student-centered environment. However, in FCs, as I transferred lecturing to outside of the classes, I saved time in class. I could identify the students’ pre-learning; then I assigned them to participate in group work and discussion sessions and enabled them to take the responsibility of problem solving and learning.* [quote from teacher interview, translated by first author].

In FCs, Ece created an interactive learning environment for students out of school in virtual classes based on the materials and the communication activities from the learning platform. In addition to audiovisual resources (videos, diagrams, graphics, tables), alternative text-based documents were shared with the students in these virtual classrooms. Ece gave her students ambitious pre-class tasks (watching videos, taking notes from the videos, questioning and answering on Edmodo, etc.) that should enhance their mathematical understanding. In this way, she could gain insight about the students’ levels of knowledge and understanding about the new topics based on their Edmodo sharings, and could dwell more on the topics in which students had expressed that they had difficulty, before or within the classroom activities. Furthermore, group work as well as individual work was regularly conducted in every lesson in FCs, and the students performed collaborative group work in four-person round-table sessions. It could be observed that Ece had difficulties in guiding the group work due to the crowded classes and a chaotic and noisy atmosphere was evident in the classroom from time to time. Ece started to use the smartboard and GeoGebra in FCs. For instance, she asked students to make drawings related to the diagrams of logarithmic functions and helped them to comprehend the relationship between exponential function and logarithmic function (see Fig. 3). Ece created a flexible environment in the class, in which students could put questions to her whenever they wanted; she trained her students through problem-solving activities and encouraged them to produce new ideas and new problem-solving strategies. Even if the solutions and ideas that students suggested were mathematically incorrect, she listened to them in the lesson and enabled them to recognize their errors by asking questions. The students, who experienced that the teacher did not judge their ideas negatively, felt in themselves more comfortable, and their belief in their ability to learn mathematics was strengthened. A student described this change as follows:

Student 1: *I did not take the floor in mathematics classes before. I sat in the back row. If I had already wanted to talk, there would not have been such an environment to do it. But in FC, everyone is engaged in mathematical activities. There was a comfortable and interactive learning environment in the class, which pleased me. I did not feel myself alone while learning mathematics. I realized that I could learn mathematics better.* [transcript from student interviews translated by first author].

#### Interaction

In her traditional teaching, Ece used to give a few examples, after explaining the topics at the blackboard and asked the students whether they had comprehended the examples, yet she did not create any opportunities in which students could make mathematical inquiries, and she did not include discussions within group work in her lessons. Ece did not allocate time for the students to think aloud on the mathematics problems and ask their questions; she shared the mathematical information directly with the students in class. The students usually did not have the chance to express their own ideas in the course of working on the mathematical problems, and they had difficulties in communicating with the teacher and peers. Additionally, there was no communication between Ece and the students outside of the lessons and students could not interact with Ece or peers about learning mathematics outside of the school.

**Fig. 2:**
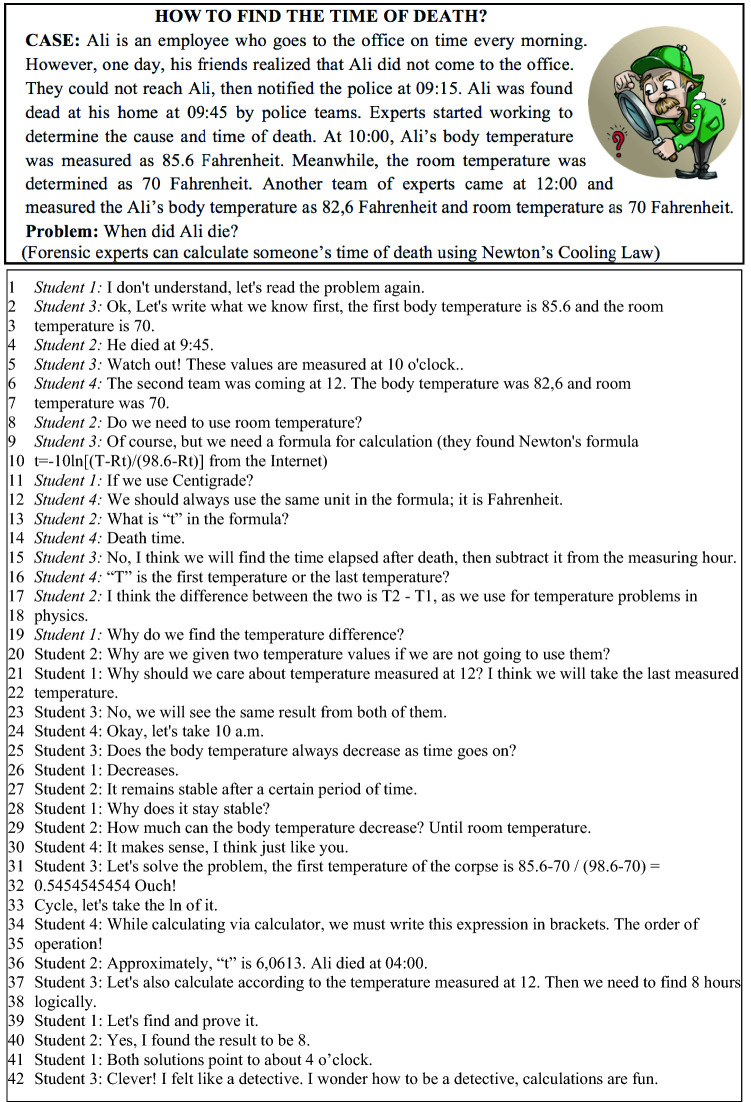
An example of mathematical problem-solving activities in the classroom and students’ dialogue within group work

In FCs, Ece and her students had a chance to communicate and interact outside of the classroom through Edmodo. She primarily encouraged her students to import their lesson-related content into the learning platform Edmodo and ask their questions there. She replied to those questions on the platform and communicated with students out of school as well. She noted down the questions that students asked on Edmodo and guided classroom discussions around these questions and a few prompt questions (examples: *What is the difference between a polynomial and a function? Do they mean the same thing? What is the degree of zero polynomial? Can all polynomials be factored?*). Ece wanted the students to develop hypotheses while solving problems and to justify their solutions using both oral and written mathematical expressions. A sample problem included in the problem-solving activities and a dialogue in one of the working groups in FCs is displayed in Fig. 2. The students worked in the groups collaboratively, generated ideas to solve the problems, shared their ideas with their peers, asked questions of each other and developed mathematical argumentations. As can be seen in Fig. 2, some of the ideas shared by the students were accepted (lines 23–24; 29–30), but some of them were refuted by other group members (lines 14–15; 21–22–23). As can be seen, students communicated well and solved the problem in an interactive way. Ece followed the students closely in the collaborative group work, listened to their ideas carefully, and guided them.

**Fig. 3:**
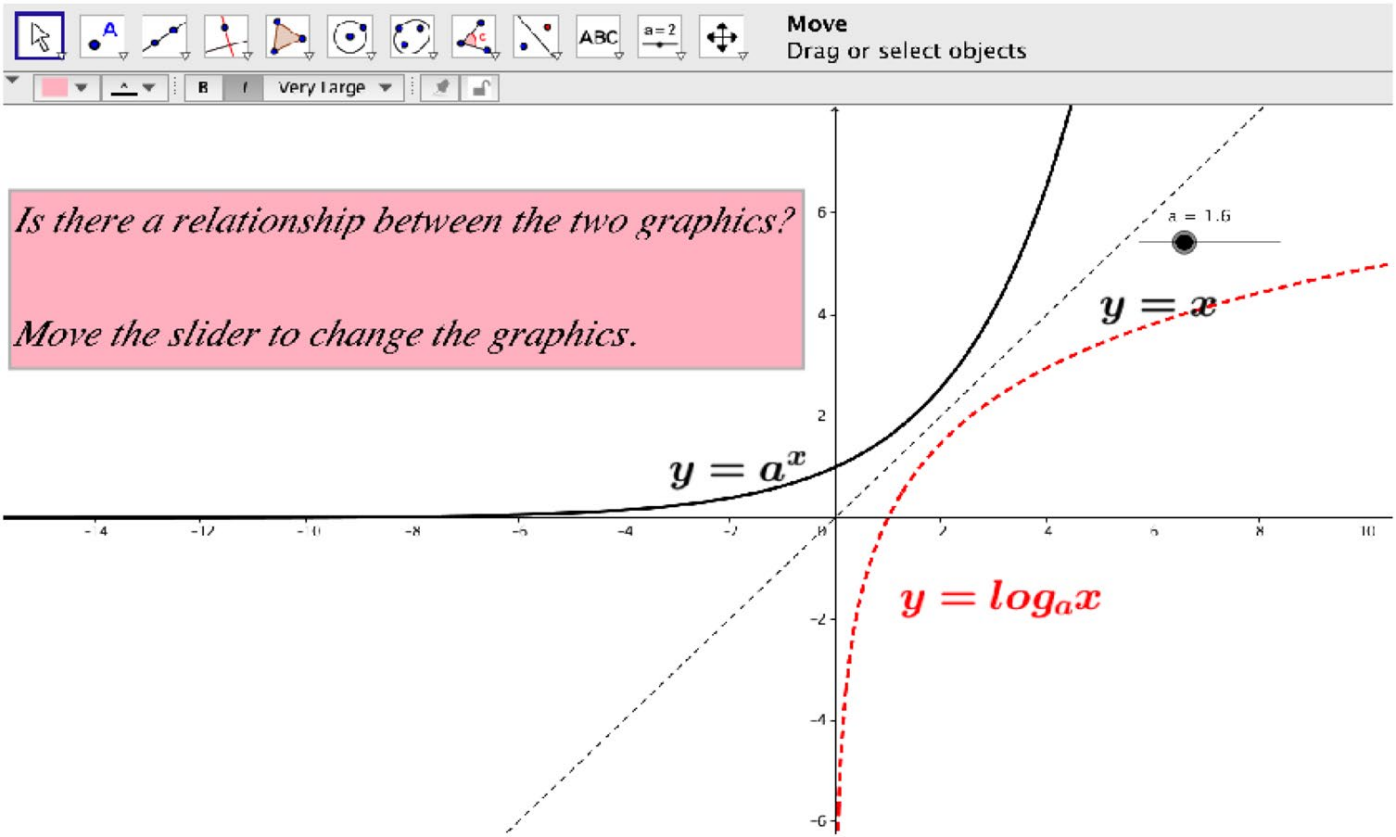
Logarithmic and exponential functions

**Fig. 4:**
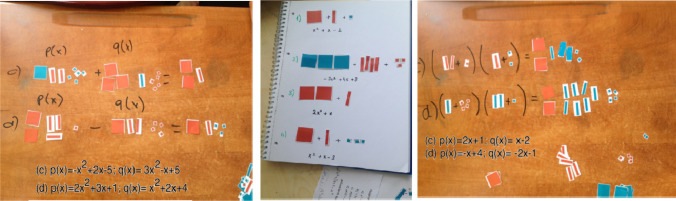
Activities regarding polynomials

Ece used technology in mathematics teaching extensively (virtual classrooms, videos, learning platform Edmodo, mathematical software GeoGebra, whiteboard, calculators, etc.) and gave students the chance to embody their ideas by engaging them in mathematical activities. Figure 3 shows a section from the activities related to logarithms that Ece performed through GeoGebra (screenshot was taken from video recordings), and in Fig. 4 (the photo was taken in group work on polynomials), a section from the students’ learning activities related to polynomials in the FCs is displayed.

#### Teacher feedback and scaffolding

Based on the classroom observations and video recordings, it could be reconstructed that Ece used direct instruction in non-FCs in the first 25–30 min of the lessons, and in the last 10–15 min, she gave algorithmic tasks to students consisting of 4–5 mathematics questions on the blackboard and then asked students to work on them. These tasks were not suitable for developing students’ mathematical thinking, but served as practice of topics and themes covered in the first part of the lesson (example: Given that p(x) = x^3^ − 7x + 5 and q(x) = x^4^ + 15x − 8, find p(x) + q(x). This is an example used by the teacher related to adding polynomials). It could be observed that neither feedback nor scaffolding was given as there was no interaction between Ece and her students in the first 25–30 min of the lessons. In the last 10–15 min of the lessons, the practice part, Ece gave the students tasks and provided feedback 4–5 times in a lesson according to student answers. Since there was not enough time available to examine all students’ solutions, she asked a few students to solve the tasks on the blackboard and gave feedback at the end of the lesson. This type of feedback was of corrective and confirmative nature. When students made mistakes in working on the task, Ece preferred to solve the task herself instead of providing scaffolding to the students. A dialogue from the interview with Ece displays the characteristic aspects of this teaching method.

*Researcher: I have observed that you usually write 4-5 mathematics questions on the blackboard in the last part of your lessons. How do you determine whether students understand the topic with them?*

*Ece: I cannot examine all students’ solutions because time is limited. I ask him or her to pick one of them and solve the problems. If there are students who have made a mistake, I make them see the correct solution on the blackboard.*

*Researcher: Do you give feedback or scaffolding to your students?*

*Ece: If the solution is correct, I say “correct”. If it is wrong, I show why it is wrong.*

*Researcher: Do you give feedback or provide scaffolding to all your students in the classroom at any time?*

*Ece: Actually, my feedback is limited. It is not possible for me to determine the needs of the students in the classroom and provide them with scaffolding. From time to time, I give homework to students to make up for their shortcomings.*

In FCs, Ece provided group work in each lesson, gave worksheets to students, and guided their work and problem solving activities closely. Students worked on mathematical tasks for 20–25 min in the classroom and attended 5–10 min of question-and-answer sessions. Ece gave individualized feedback to her students, including explanations, hints, encouragement, support, inquiry, confirmation, and correction. In each lesson, Ece followed the students in their group work and gave immediate feedback to them, whenever it was needed. The difference in the quantity of feedback given by Ece in the two kinds of teaching approaches is a strong indicator of the changes in her teaching method: in a non-FC lesson, she gave feedback 4–5 times, compared to 45–55 times in a FC lesson. Ece did not provide scaffolding at all in non-FCs, she provided it 7–8 times in FCs. Students used a flexible and an interactive learning environment in FCs: they immediately contacted Ece when they needed scaffolding or feedback. In this way, Ece was able to allocate more time to the students who needed more support. 88% of the students stated in the students’ questionnaire that they had received more feedback and scaffolding in FCs and they had understood the topics better due to the teacher feedback and scaffolding. In FCs Ece also gave feedback to her students outside of school with the help of Edmodo. Students who had watched the videos could ask questions of Ece in virtual classrooms, and Ece provided corrective, confirmative, and explanatory feedback to help her students. Table 3 shows the characteristics of scaffolding and feedback given by Ece in non-FCs and in FCs.

**Table 3:** Characteristics of teacher’s feedback and scaffolding in FCs and non-FCs

Feedback and scaffolding	Non-FCs		FCs	
	Out-of-class	In-class	Out-of-class	In-class
Frequency^a^	–	Feedback 4–5 times scaffolding	Feedback 10–15 times scaffolding	Feedback 35–40 times scaffolding 7–8 times
Type^b^	–	Corrective, confirmative	Corrective, confirmative, explanatory	Confirmative, corrective, encouraging, explanatory, hinting, inquiring, and supportive
Timing	–	End of the class hours	In Q&A sessions	Homogeneously distributed

^a^For a 40-min lesson, ^b^feedback type

#### Assessment strategies

For non-FCs, Ece stated that she had graded her students according to two separate mathematics examinations held in one semester; in other words, she applied static assessment methods. Examination scores were regarded as the only achievement criteria, and the students who attained an average of 50% and higher in these scores were accepted to receive the passing grade in the course. Ece did not take into account the students’ mathematical learning processes apart from these scores. The parents were informed of their children’s academic standing in parent–teacher meetings generally held once per semester.

In FCs, Ece observed her students’ learning activities more closely, provided them with personal feedback and scaffolding, allowed them to speak in the lesson, listened to their ideas, and gave them two additional quizzes that comprised 20 multiple-choice questions on the topics covered in the lecture videos, in addition to the compulsory examinations. However, she did not change her grading style and determined the students’ mathematics achievement solely according to the marks in the compulsory exams as in non-FCs. Different from non-FCs, in FCs, parents could communicate with the teacher directly and receive immediate feedback about the students’ academic standing as they could log in to the learning platform Edmodo with a parent account. The reason why Ece did not apply a dynamic assessment approach in FCs was examined within the interview with Ece. She expressed her concerns that the changing of her teaching approach was already hazardous for her and that changing the assessment method at the same time might have caused unpredictable strong reactions from students and parents. As she wished to avoid community pressure (from students, parents, and school administration) she did not consider alternative dynamic assessment methods. She added that, while mathematics educators at universities were flexible in their courses, teachers of K–12 levels were under pressure from the triad: parents, students, and administrators. Apparently, Ece felt intimidated by the probable reactions and indeed could not completely implement the innovations she had planned into action as became clear in the following statement in the interview with her:

Ece: *I underwent some training on active teaching methods, but the reality in practice is not as the one in theory. Power at the K–12 level in Turkey is in the hand of parents, students, and administrators instead of teachers, unfortunately. They sometimes attempt to interfere in even what and how you are lecturing in the class. Therefore, lots of teachers who have begun the job as idealists may become confined to traditional approaches.* [transcript from teacher interview, translated by first author].

## Summary of the key results

The results of the study point out that FCs provide challenges and opportunities for teaching in mathematics education. With reference to the challenges of FCs, Ece, who underwent a change in her teaching routine, stated that she felt uneasy and incompetent at the beginning of the FC implementation. She thought that if she was not successful in teaching using FCs, the good teacher impression that she had made on the students would be affected negatively, and thus she felt nervous. She also indicated that designing the FCs and producing content took lots of time and was demanding and that she needed the knowledge of how to use certain software in order to create high-quality lecture videos and edit them properly. She found it boring and exhausting to be constantly checking whether the students had watched the lecture videos and complained that the time she allocated for her social life got restricted when she kept in touch with all the students outside of school and needed to follow their development after class-time as well. Since the classrooms were crowded, it could be observed that sometimes a chaotic (noisy) atmosphere prevailed in the classes, and Ece was challenged in monitoring the students and their learning processes. It became a difficult task for Ece to involve two students who refused the change of the teaching approach. Ece stressed that she did not consider teaching with FC techniques in the future because of all these difficulties.

FCs provided some opportunities as well as challenges in terms of the teacher. In this vein, Ece gained experience in FCs and expanded her knowledge on innovative teaching approaches. She reflected that her job satisfaction increased as she made a great effort in teaching mathematics in the FCs. Ece had allocated enough time in the FCs for active learning activities that she could not implement in non-FCs. She also accomplished the integration of technology in teaching mathematics in the FCs by use of the Internet, Edmodo, GeoGebra, lecture videos, smartboard, calculators, mobile devices, and online resources. Ece was able to identify missed learning progress by closely monitoring students’ learning activities in the FCs; she could give them feedback and scaffolding in a timely way. Because of FC, Ece increased the level of communication and interaction with her students and discovered more information about their mathematical learning activities outside the classroom.

## Discussion, conclusion and limitations

The results of this case study are limited to the fact that only one teacher’s teaching experiences were analyzed, although the study comprised activities in two FCs and two non-FCs covering 8 weeks. The teacher was a well-educated (doctoral candidate) mathematics teacher with 6 years of professional experience; however, her FC experiences were limited to 2 weeks of practice in the pilot study. These limitations have to be taken into account when interpreting the results. The main results of this case study highlighted that FC has the potential to transform mathematics teaching fundamentally, as the teacher changed her teaching in secondary mathematics classrooms quite considerably. The central elements of teaching—*teaching and learning environments, interaction mode, feedback and scaffolding—*provided by the teacher underwent a radical change through FC in accordance with the perspectives of social constructivism (Vygotsky 1978). However, the teacher did not achieve a shift in her *assessment* methods, although she applied flipped teaching strategies in her classes. One reason the teacher gave was the fear that a failed FC implementation might produce an unfavorable reaction from the triad of students, parents, and school administrators. These concerns and emphasis point out that—at least in countries with a strong influence of educational administration—teachers are not free in the design of their teaching. Overall, this aspect highlights that there are paradigmatic obstacles in the transition from traditional teaching methods to innovative ones such as FC; amongst others, an obstacle is the need for change in the existing values and beliefs of stakeholders of education (Melville et al. 2013).

In FCs, the teacher managed to create an interactive and flexible teaching and learning environment, with students being engaged in mathematical activities in an interactive way, starting from pre-class activities. Students had plenty of work in FCs, such as watching the lecture videos, making sense of the videos, participating in Q&A activities in Edmodo, engaging in problem-solving activities and discussion sessions, and working collaboratively as well as independently on mathematical problems. The teacher managed to engage students in discourses on mathematical tasks and learning activities in FCs and encouraged them to communicate effectively. These attempts motivated students to think aloud about mathematical problems and tasks and to discuss their ideas in a relaxed atmosphere. Their mathematical communication skills improved, and they interacted with the teacher and their peers while working in groups. The flexible and interactive environment offered by FCs made students more interested in mathematics and contributed to their problem-solving skills. To summarize, these results showed that in line with the constructivist teaching-and-learning approach, the teacher managed to encourage students to be more active and engaged in FCs and enabled the students to foster their own construction of knowledge in social interaction and collaboration. Students could develop their own mathematical thinking by interacting with their teachers and by collaboration in classrooms (Kim 2001; Vygotsky 1978). The teacher exploited technology (smartboard, calculators, GeoGebra, Edmodo, online sources, videos, Internet) as a tool in the physical classroom as well as in the virtual classroom, in order to allow a differentiated teaching approach and an interactive teaching environment. In FCs, the teacher was able to follow the students’ work closely, to provide immediate feedback on students’ learning performance, and to support students’ mathematical understanding with scaffoldings. This approach is in line with the social constructivist theory, where the role of the teacher is guiding students’ work and assisting students to develop their own solutions, not prescribing the solution (Schreiber and Valle 2013); good learning occurs in ZPD (Vygotsky 1978) by means of scaffolding. In principle FCs allow teachers to follow students’ learning activities and observe their reactions; thus, teachers can better understand and meet students’ needs (Bergmann and Sams 2012; Cevikbas and Argün 2017; Chen and Wen 2019). Through the online learning platform used, the teacher learned more about her students’ out-of-class learning activities as well as in-class activities and customized her scaffolding and feedback strategies. Teacher feedback provides students with insight into their learning performance (Cevikbas and Argün 2016) and scaffolding offers students opportunities for their cognitive development. The teacher invested a high amount of work in order to improve students’ mathematical capacity by feedback and scaffolding in FC implementations. These efforts also strengthened her job satisfaction and professional development. Although she closely followed the students’ learning activities, she assessed students through a static assessment method. It was assumed that using this type of assessment method in FCs might have negative effects on students’ engagement in mathematical activities; thus, dynamic assessment approaches were considered to be more appropriate to evaluate FC activities. In a Vygotskian perspective, traditional static assessment strategies portray the student’s level of actual development, and dynamic assessment strategies uncover the student’s potential level of development (Palincsar 1998). From this perspective, it can be stated that the teacher failed to evaluate students’ potential development levels. We can consider that in this specific case, changing the assessment strategy was more difficult than changing the teaching method. More emphasis should be placed on this issue in future studies.

At a technical level, we identified that the length of the lecture videos in flipped secondary mathematics classrooms can be between 10 and 20 min, based on a pilot study and a teacher’s teaching experiences in FCs. The ideal length of lecture videos can vary, depending on different age groups. Some researchers recommended that the length of videos be less than 15 min (Bergmann and Sams 2012) or about 10 min (Chen and Wen 2019). Videos should incorporate prompt questions, examples with solutions, and lecturing, all of which contribute to students’ mathematical thinking and development of mathematical meaning. The students preferred that the instructor in the videos be their own teacher, not any other. Furthermore, employing videos shared on different online platforms or social media channels that are not reviewed by any mathematics educator can cause problems concerning the validity of the content. Additionally, the adequate relation between the topic shared in the lecture videos and in-class activities was the critical point for the basic FC design. In this study, we found that FCs presented challenges as well as opportunities in teaching mathematics. The uncertainty of teaching in FCs for the first time made our teacher feel uncomfortable. She mentioned that it was troublesome and time-consuming to produce the content in the FCs. The study of Lo and Hew (2017) reported similar results and pointed out that the crucial issues in FCs included teachers’ heavy workload in creating content. The teacher also had difficulties in guiding the group work and wasted a lot of energy due to the large class size. As the constant contact with students outside the classroom produced a high amount of work, it is recommended that teachers and students should determine a certain period for out-of-school communication and interactive discussions on learning platforms in order to maintain a work–life balance. Although there were several opportunities for teaching mathematics in FCs, our teacher stated that she would not consider teaching mathematics in FCs in the future because of the difficulties mentioned above.

Overall, FC has the potential to change the paradigm of teaching mathematics and inspire teachers to generate new ideas and gain new educational experiences. However, it is necessary for teachers to consider whether they and their students are ready for FCs and whether they have the families’ and administrators’ support. Although teaching mathematics in FC has some difficulties, well-designed FCs presents a great opportunity to boost students’ mathematical thinking and understanding using differentiated teaching in interactive environments. To conclude, teachers who are mentally and technically ready to transform mathematics classrooms into FCs can strongly develop students’ mathematical capacity in FCs. The results of the research revealed that FC is not a panacea (Lo and Hew 2017), but it can contribute to mathematics education. Further research should focus on the impact of teacher characteristics on the success of the introduction of FCs, such as professional experience, teaching experience in FCs, competence in using kinds of required technology, and educational background. Teachers who are more experienced with technology in teaching than the teacher in our study, may give their students more effective feedback, may provide stronger scaffolding and more differentiation in their teaching. Additionally, future studies may shed light on the role of technology in teaching mathematics in FCs. In this study, our teacher used technology as a tool to make her teaching differentiated and more interactive, but of course technology can provide many more possibilities for enriching the mathematics classrooms.

## References

[CR1] Ahmed HOK (2016). Flipped learning as a new educational paradigm: An analytical critical study. European Scientific Journal.

[CR2] Bagley S (2020). The flipped classroom, lethal mutations, and the didactical contract: A cautionary tale. Primus.

[CR3] Baker, J. W. (2000, April). *The “classroom flip”: Using web course management tools to become a guide by the side*. Paper Presented at the 11th International Conference on College Teaching and Learning*.* Jacksonville, FL.

[CR4] Beatty R, Geiger V (2009). Technology, communication, and collaboration: Re-thinking communities of inquiry, learning and practice. Mathematics education and technology-rethinking the terrain.

[CR5] Bergmann J, Sams A (2012). Flip your classroom: Reach every student in every class every day.

[CR6] Bishop, J. L., & Verleger, M. A. (2013, June). The flipped classroom: A survey of the research. In *ASEE National Conference Proceedings, Atlanta, GA* (Vol. 30, No. 9, pp. 1–18).

[CR7] Brown AF, Mehring J, Leis A (2018). Implementing the flipped classroom: Challenges and strategies. Innovations in flipping the language classroom.

[CR8] Cevikbas, M. (2018). *Ters-yüz sınıf modeli uygulamalarına dayalı bir matematik sınıfındaki öğrenci katılım sürecinin incelenmesi.* Unpublished Doctoral Dissertation, Gazi University.

[CR9] Cevikbas M, Argün Z (2017). An innovative learning model in digital age: Flipped classroom. Journal of Education and Training Studies.

[CR10] Cevikbas M, Argün Z (2016). Matematik öğretmenlerinin yanlış cevaplara verdikleri dönütlerin öğrenci özsaygıları üzerindeki rolü. Gazi Egitim Fakültesi Dergisi.

[CR11] Chen LL (2016). Impacts of flipped classroom in high school health education. Journal of Educational Technology Systems.

[CR12] Chen, F., & Wen, F. (2019). Research on Flipped Classroom teaching mode of high school bmathematics under the background of “Internet+”. In *3rd International Conference on Education, Management Science and Economics*. Atlantis Press.

[CR13] Cockrum T (2014). Flipping your English class to reach all learners: Strategies and lesson plans.

[CR14] Creswell JW (2013). Qualitative inquiry and research design; Choosing among five approaches.

[CR15] Davies RS, Dean DL, Ball N (2013). Flipping the classroom and instructional technology integration in a college-level information systems spreadsheet course. Educational Technology Research and Development.

[CR16] Engelbrecht J, Llinares S, Borba MC (2020). Transformation of the mathematics classroom with the internet. ZDM Mathematics Education.

[CR17] Flipped Learning Network (2014). Definition of flipped learning. https://flippedlearning.org/definition-of-flipped-learning/. Accessed 2 Sep 2020.

[CR18] Fredriksen H (2020). Exploring realistic mathematics education in a flipped classroom context at the tertiary level. International Journal of Science and Mathematics Education.

[CR19] Fulton KP (2012). 10 reasons to flip. Phi Delta Kappan.

[CR20] Goos M, O’Donoghue J, Ní Ríordáin M (2020). Designing a national blended learning program for “out-of-field” mathematics teacher professional development. ZDM Mathematics Education.

[CR21] He J (2020). Research and practice of flipped classroom teaching mode based on guidance case. Education and Information Technologies.

[CR22] Heid K, Masalski WJ (2005). Technology in mathematics education: tapping into visions of the future. Technology-Supported Mathematics Learning Environments: NCTM 67th Yearbook.

[CR23] Hooks M (2015). Pinterest: A tool for lesson planning. Mathematics Teacher.

[CR24] Jarvis W, Halvorson W, Sadeque S, Johnston S (2014). A large class engagement( LCE) model based on service-dominant logic (SDL) and flipped classrooms. Educational Research and Perspectives.

[CR25] Jian K (2020). Reform and innovation of ideological and political course teaching from the perspective of flipped classroom. Advances in Higher Education.

[CR26] Joubert J, Callaghan R, Engelbrecht J (2020). Lesson study in a blended approach to support isolated teachers in teaching with technology. ZDM Mathematics Education.

[CR27] Kim, B. (2001). Social constructivism. In M. Orey (Eds.), *Emerging perspectives on learning, teaching, and technology.* https://www.researchgate.net/profile/Beaumie_Kim. Accessed 6 Sep 2020.

[CR28] Lage MJ, Platt GJ, Treglia M (2000). Inverting the classroom: A gateway to creating an inclusive learning environment. The Journal of Economic Education.

[CR29] Lagrange JB, Kynigos C (2014). Digital technologies to teach and learn mathematics: Context and re-contextualization. Educational Studies in Mathematics.

[CR30] Lavicza Z (2010). Integrating technology into mathematics teaching at the university level. ZDM.

[CR31] Lo CK, Hew KF (2017). A critical review of flipped classroom challenges in K–12 education: Possible solutions and recommendations for future research. Research and Practice in Technology Enhanced Learning.

[CR32] Longman. (2020). *Longman dictionary of contemporary English.* https://www.ldoceonline.com/dictionary/reform. Accessed 31 Aug 2020.

[CR33] Marzouki OF, Idrissi MK, Bennani S (2017). Effects of social constructivist mobile learning environments on knowledge acquisition: A meta-analysis. International Journal of Interactive Mobile Technologies.

[CR34] Maxwell JA (2013). Qualitative research design: An interactive approach.

[CR35] Mazur AD, Brown B, Jacobsen M (2015). Learning designs using flipped classroom instruction. Canadian Journal of Learning and Technology.

[CR36] Merriam SB, Tisdell EJ (2016). Qualitative research: A guide to design and implementation.

[CR37] McGivney-Burelle J, Xue F (2013). Flipping calculus. Primus.

[CR38] Melville W, Kajander A, Kerr D, Holm J (2013). Uncertainty and the reform of elementary math education. International Scholarly Research Notices.

[CR39] Miles MB, Huberman AM (1994). Qualitative data analysis.

[CR40] National Council of Teachers of Mathematics (2011). *Strategic use of technology in teaching and learning mathematics.* https://www.nctm.org/Standards-and-Positions/Position-Statements/Strategic-Use-of-Technology-in-Teaching-and-Learning-Mathematics/last. Accessed 25 July 2020.

[CR41] Olive, J., Makar, K., Hoyos, V., Kor, L. K., Kosheleva, O., & Sträßer, R. (2009). Mathematical knowledge and practices resulting from access to digital technologies. In *Mathematics education and technology-rethinking the terrain* (pp. 133–177). Boston: Springer.

[CR42] Palincsar AS (1998). Social constructivist perspectives on teaching and learning. Annual Review of Psychology.

[CR43] Schmidt SM, Ralph DL (2016). The flipped classroom: A twist on teaching. Contemporary Issues in Education Research.

[CR44] Schreiber LM, Valle BE (2013). Social constructivist teaching strategies in the small group classroom. Small Group Research.

[CR45] Thurlings M, Vermeulen M, Bastiaens T, Stijnen S (2013). Understanding feedback: A learning theory perspective. Educational Research Review.

[CR46] Trigueros M, Sandoval I, Lozano M (2020). Ways of acting when using technology in the primary school classroom: contingencies and possibilities for learning. ZDM Mathematics Education.

[CR47] Voigt M, Fredriksen H, Rasmussen C (2020). Leveraging the design heuristics of realistic mathematics education and culturally responsive pedagogy to create a richer flipped classroom calculus curriculum. ZDM Mathematics Education.

[CR48] Vygotsky LS (1978). Mind in society: The development of higher psychological processes.

[CR49] Wood D, Bruner JS, Ross G (1976). The role of tutoring in problem-solving. Journal of Child Psychology and Psychiatry and Allied Disciplines.

